# The Lighter Touch: Less-Restriction in Sequentially Implemented Behavioral Sleep Interventions for Children with Rare Genetic Neurodevelopmental Conditions

**DOI:** 10.1007/s10803-024-06234-4

**Published:** 2024-02-07

**Authors:** Emma C. Woodford, Laurie K. McLay, Karyn G. France, Neville M. Blampied

**Affiliations:** 1 Faculty of Health, Te Kaupeka Oranga, University of Canterbury, Christchurch, New Zealand; 2 Psychology Speech & Hearing, Faculty of Science, Te Kaupeka Pūtaiao, University of Canterbury, Christchurch, New Zealand; 3 Child Wellbeing Research Institute, University of Canterbury, Christchurch, New Zealand

**Keywords:** Sleep, Behavioral interventions, Least restrictive, Rare genetic disorders, Neurodevelopmental disorders

## Abstract

**Purpose:**

The prevalence of sleep difficulties among children with rare genetic neurodevelopmental conditions (RGNC) is high. Behavioral interventions are commonly used in the treatment of sleep difficulties in children with neurodevelopmental conditions such as autism, however, research is scarce in children with RGNC. The range of co-occurring complexities within this population, means there is a need for research to not only determine the effectiveness of behavioral sleep interventions, but also which components might be the least restrictive (i.e., intensive/aversive) and minimally sufficient.

**Methods:**

This study used a single-case multiple baseline design to investigate the effectiveness and acceptability of behavioral sleep interventions, indicated within a Functional Behavior formulation in eight children with RGNC (*M* = 7.3 years). Intervention components were sequentially administered across up to three phases, based on the principle of less restriction (from least to relatively more intensive) to determine what might be minimally sufficient.

**Results:**

Results showed an improvement in sleep onset latency, night wakings, early morning waking and unwanted bed-sharing for 7/7, 6/7, 3/3 and 3/3 children respectively. Improvement was observed for most participants following the less restrictive phases of intervention (circadian modifications, antecedent modifications and positive reinforcement), however, more restrictive, albeit modified, extinction procedures were still implemented for five participants. Improvements were maintained at follow-up and interventions were deemed acceptable to parents.

**Conclusions:**

Less restrictive function-based behavioral strategies are an effective, and in some cases sufficient, contribution to a sequence of interventions for a range of sleep difficulties. They should be implemented first, before more restrictive strategies.

**Supplementary Information:**

The online version contains supplementary material available at 10.1007/s10803-024-06234-4.

## Introduction

Rare Genetic Neurodevelopmental Conditions (RGNC) represent heterogenous syndromes arising from chromosomal alterations affecting fewer than one in 2000 people (European Commission, 2021; e.g., 22q11.2/Di George (22q), Angelman, Smith-Magenis and Phelan-McDermid syndromes). Individuals with RGNC experience a range of difficulties including developmental and intellectual delay, medical complexities, and sleep difficulties (McLay et al., 2019; Neo et al., 2021).

Sleep difficulties affect up to 90% of children with RGNC (Agar et al., 2021; Angriman et al., 2015; Kronk et al., 2010) and seldom resolve without effective intervention. These can adversely affect daytime behavior and well-being of children and their families (Kronk et al., 2010; Mörelius & Hemmingsson, 2014) meaning that successful intervention can enhance child development and family functioning (McLay et al., 2022a). The etiology of sleep disturbance in RGNC is multifaceted, with medical (e.g., seizures, gastrointestinal issues; Ghanizadeh & Faghih, 2011), biological (e.g., altered melatonin regulation; Woodford et al., 2021), psychological (e.g., anxiety and attention deficit hyperactivity disorder; Konjarski et al., 2018) and behavioral (e.g., inadvertent reinforcement of sleep-interfering behavior; McLay et al., 2022b) factors all identified as potential causes. Despite the known interactions between these biopsychosocial causes, sleep intervention for children with RGNC has largely focused on pharmacological and medical approaches. While medications for sleep (e.g., melatonin) and/or co-occurring conditions interfering with sleep have proven to be effective in the treatment of sleep difficulties (e.g., Bruni et al., 2019), they are not without limitations. Of note, medications do not target the behavioral factors that may give rise to, exacerbate, and maintain sleep difficulties (Blampied & Bootzin, 2013; McLay et al., 2022b).

In response to children’s behavioral sleep difficulties parents/caregivers (henceforth, parents) draw on a range of strategies including altering the physical environment, comforting, ignoring, scolding, providing or removing tangible items and so forth (Johnson & McMahon, 2008; Shetty et al., 2022). Some of these may help reduce sleep difficulties, while others may inadvertently exacerbate them as well as the child’s distress. Behavioral sleep interventions allow parents to choose strategies which involve modification to the antecedent and consequence variables that maintain both sleep-facilitative and sleep-interfering behavior in a manner maximising the likelihood of positive change (Blampied, 2013; Blampied & Bootzin, 2013; McLay et al., 2022b). Antecedent modifications may include enhancing motivation to sleep by increasing homeostatic sleep pressure (i.e., the drive for sleep) through sleep/wake rescheduling (i.e., circadian modifications; Ford et al., 2021; Schreck, 2022; Woodford et al., 2022); changes to the sleep environment to improve sleep hygiene; and provision of discriminative stimuli that guide the completion of the bedtime routine so the individual achieves a state of behavioral quietude that permits transition to sleep. Consequence modifications may include reinforcement of sleep-facilitative behavior (i.e., rewards) and the removal of reinforcement for sleep-interfering behavior such as parent attention and access to preferred items, an application of behavioral extinction (Carnett & McLay, 2022; France & Blampied, 2005; McLay et al., 2021; van Deurs et al., 2021). Such interventions have a strong evidence-base for the treatment of insomnia (i.e., difficulties initiating and maintaining sleep) in neurotypical children (Meltzer & Mindell, 2014); an emerging evidence-base for children with higher incidence neurodevelopmental conditions (e.g., autism; McLay et al., 2021; Rigney et al., 2018); and a minimal evidence-base for children with RGNC (McLay et al., 2019).

To identify relevant antecedent and consequence variables and thus understand what to modify therapeutically, Functional Behavioral Assessment (FBA) is often used (McLay et al., 2022b). FBA provides essential information for an individualized case formulation and the design of evidence-based interventions, largely implemented by parents (Carnett & McLay, 2022; France, 2022). FBA has been used extensively to inform the selection of behavioral sleep interventions for children with neurodevelopmental conditions such as autism and has been shown to enhance the efficacy of interventions (McLay et al., 2021, 2022b; van Deurs et al., 2021, Jin et al., 2013). However, research with children with RGNC is extremely limited. Only 3/9 studies identified by McLay et al. (2019) to have evaluated the effectiveness of behavioral sleep interventions with such children utilised FBA to inform the selection of strategies (Curfs et al., 1999; Didden et al., 1998; Weiskop et al., 2005).

While behavioral sleep interventions have been reported to be effective, most have included multiple components administered concurrently, meaning that the effectiveness of individual components remains undetermined. This prevents identification of what may be minimally sufficient for change (Sanders et al., 2014). Furthermore, these multi-component interventions frequently included extinction or modified extinction procedures which have been reported to be less acceptable to families for several reasons (Etherton et al., 2016; Woodford et al., 2022). Of note, these procedures are generally perceived to cause higher rates of parent and child distress and to be at odds with particular parenting values or practices (e.g., responsive parenting; Ainsworth, 1969). In addition, they may result in temporary worsening of the sleep-interfering behavior (i.e., a response burst; France & Blampied, 2005) before an improvement is observed. This can be difficult for parents to manage, leading to further distress, reduced treatment integrity, and even drop-out (Etherton et al., 2016).

Relevant to the selection of interventions is the principle of *least restriction*, where interventions may be ranked on a dimension where restrictive means stressful for those involved; intensive in that they require extended effortful actions from those concerned; and/or intrusive in that they affect family routines (Blampied & van Deurs, 2022; Byskov, 2019; Johnston & Sherman, 1993). It is seen as ethical to first select the least restrictive intervention consistent with achieving effective change and only move to more restrictive interventions if they are needed to achieve important therapy objectives (Byskov, 2019; Childress et al., 2002; Johnston & Sherman, 1993). Any intervention that is both least restrictive and effective may be considered a minimally sufficient intervention (Sanders et al., 2014). Finding such interventions for children and families affected by RGNC is important to foster the child’s wellbeing and, particularly, to reduce the demands and stress placed on the family system, which may already be high due to the demands of caring for a child with RGNC (France et al., 2022; McLay et al., 2022a; Mörelius & Hemmingsson, 2014).

A prior study conducted by the authors (Woodford et al., 2022) evaluated the feasibility of using the principle of less restriction in the context of FBA-informed treatment planning, and its practical applicability to sleep disturbance in children with neurodevelopmental conditions including RGNC. Most participants received multi-component interventions made up of a range of less restrictive strategies including circadian, antecedent, and positive reinforcement procedures. Results were promising, with an improvement noted across a range of sleep difficulties with varying functions. However, this study did not permit researchers to determine what might be both less restrictive *and* minimally sufficient for positive change.

There are currently no clear guidelines specifying the least to most restrictive behavioral sleep interventions for any population, thus, for this study, well-established, behavioral pediatric sleep interventions such as those identified above, were selected for potential use, grouped and then ranked a priori from least to most restrictive as follows: 1) Circadian modifications (Schreck, 2022), p 2) other antecedent modifications (Ford et al., 2021; McLay et al., 2021), p 3) use of positive reinforcement (McLay et al., 2021), and p 4) modified extinction (Carnett & McLay, 2022). This ranking was based on what might be experienced by both the parent and child as least stressful, complex, effortful, and intrusive. Within this rationale we consider that circadian modifications may increase homeostatic sleep pressure at night, and thereby reduce a range of sleep difficulties such as sleep onset latency (SOL) and night waking (NW). In addition, these interventions are generally relatively simple to understand and implement, making them the likely least restrictive options. Other antecedent modifications including sleep hygiene, visual prompts and stimulus control strategies come next in the order, as they require more changes to the sleep routine and environment. These are then followed closely by positive reinforcement which provides motivation for the child to engage in sleep-facilitative behavior (e.g., independent settling) but can be difficult to implement within the sleep context given the delay between sleep onset and receiving reinforcement on waking. In addition, rewards can be arousing when received before sleep onset, diverting the child from establishing behavioral quietude and ultimately delaying sleep onset (Blampied & van Deurs, 2022). Although positive reinforcement may be considered more restrictive than antecedent options, in this study they were combined, in consideration of the outcomes of the FBA and family preferences. Parents often supported a combined approach due to the complementary nature of antecedent modifications and positive reinforcement systems (e.g., use of positive reinforcement for completion of the bedtime routine, or explanation of how the child might achieve a reward in a Social Story). Extinction procedures are considered more restrictive because they are generally aversive for both the parents and child, and when modified with parental presence to be less aversive, are complex and effortful for parents to implement (France & Blampied, 2005). Consequence modifications involving punishment of any kind, or unmodified extinction, were not considered or used for ethical reasons.

Given a range of sleep intervention strategies consistent with an individual’s FBA and ordered by restrictiveness, research is needed to examine how minimal sufficiency might be achieved, moving to more restrictive interventions only if less restrictive ones have not produced sufficient positive change. Therefore, the aims of this study were to investigate (a) if minimally sufficient interventions can be identified for sleep difficulties in children with RGNC, (b) the maintenance of any effects at 4–6 and 10–14 weeks post-intervention, and (d) the acceptability of the interventions to parents. The overall aim was to demonstrate how FBA and the principle of less restriction might be combined in intervention planning.

## Method

### Participants

Research was approved by the relevant university Human Ethics Committee. Participants were recruited from throughout New Zealand via service providers for children with RGNC. Parents were provided details of the study in a screening phone call and were emailed information and consent/assent forms. Informed consent was obtained from all parents and child assent was sought where child capacity permitted. Consent forms were returned digitally, and parents could ask questions via phone or email.

Participants who approached the research team then met the following inclusion criteria: (a) were between 18 months–21 years of age; (b) had a diagnosis of RGNC as verified by genetic testing or were undergoing genetic assessment (Emily); and (c) had parent-reported sleep difficulties confirmed during assessment, were accepted into the study. If the child had a co-occurring condition not currently managed or that might interfere with the implementation of a behavioral sleep intervention (e.g., nocturnal seizures, tube feeding), approval was obtained from the child’s primary physician, otherwise they were excluded for safety reasons. Eight children (three female and five male; pseudonyms used) between the ages of 4–14 years met inclusion criteria. RGNC diagnosis varied across children. Table 1 summarizes participant characteristics.

**Table 1:** A summary of participant characteristics at commencement of intervention

Participant	Age at intake (Y-M)	Gender	VABS-III communication age equivalence (Y-M)	Primary diagnosis	Co-occurring conditions	Medications
Carl	14–7	M	*Rec:* 3–6 *Exp:* 3–4	Smith-Magenis syndrome	ID; scoliosis	Atenolol; Iron; Melatonin; Panadol (for back pain)
Greg	9–7	M	*Rec:* 2–0 *Exp:* 2–8	Jordans syndrome	Epilepsy; hypotonia; ID	Clobazam; Lamotrigine
Emily	8–4	F	*Rec:* 1–6 *Exp:* 2–6	Suspected genetic syndrome (undergoing testing)	ADHD; ASD; epilepsy; GDD; sinus issues	Levetiracetam; Melatonin; Ritalin
Liam	7–4	M	*Rec:* 1–0 *Exp:* 0–3	WDR45 disorder	Epilepsy; ID	Levetiracetam
Polly	7–1	F	*Rec*: 3–2 *Exp:* 3–11	22q13.3/Phelan-McDermid syndrome	Epilepsy; gastrointestinal issues; GDD; hypotonia	Melatonin
Courtney	5–1	F	*Rec:* 3–3 *Exp:* 4–4	Dup 15q syndrome	GDD; hypotonia; reflux	Melatonin; Molaxole; Omeprazole
Henry	4–6	M	*Rec:* 0–9 *Exp:* 0–5	22q11.2/ DiGeorge syndrome	Gastrointestinal issues; GDD; low motility; visual impairment	Molaxole; Omeprazole
Jack	4–6	M	*Rec:* 2–9 *Exp:* 4–8	22q11.2/ DiGeorge syndrome	GDD	Melatonin

Pseudonyms used

*Exp* Expressive language, *F* Female, *GDD* Global Developmental Delay, *ID* Intellectual Disability, *M* Male, *Rec* Receptive language, *VABS-III* Vineland Adaptive Behavior Scales, third edition, *Y-M* Years-Months

### Study Design

Intervention outcomes were evaluated using a single-case non-concurrent multiple baseline across-participants design with random allocation of pre-specified baseline lengths. This made it possible to determine if there was a replicable effect across specific behaviors when and only when intervention was introduced, reducing the probability that change was related to extraneous variables and supporting the inference that there was an effect (Barlow et al., 2009).

### Setting

All therapeutic support was provided remotely via email, phone, and video conferencing to accommodate national recruitment. All interventions were implemented by parents, in the family home, with the support of the research team.

### Measures

#### Sleep Diaries

Paper or electronic sleep diaries were completed by parents daily, across study phases. Parents recorded information about their child’s sleep setting; the timing, frequency, and duration (minutes) of daytime naps and NW; SOL (minutes from bedtime to initial sleep onset); frequency of curtain calls (CCs; bids for parent attention before initial sleep onset); time spent bed-sharing (child in parents bed or parents in child’s bed; calculated for visual analysis as a percentage of time spent bed sharing relative to total time in bed); and morning waketime which was used to calculate the duration of early morning waking (EMW; minutes awake prior to the family-set acceptable waketime) for participants for whom it was an intervention target. Parents also recorded information about their child’s sleep-interfering behavior and their response. Key dependent variables were extracted from sleep diary data for visual analysis for all but one participant where video recording was the primary data source (see below).

#### Video Recordings

TP-link Tapo C100 night vision Wi-fi cameras with micro-SD cards and written operating instructions were provided to families. Nightly video recordings were primarily used to gather interobserver agreement data (IOA), however, for Carl, it was the primary source of data as his sleep difficulties were not always detectable to his parents and were unable to be accurately recorded in diaries. For Greg, video was used to complete information missing from long-term follow-up (LTFU) sleep diaries. Video of the child’s sleep was coded for a minimum of 20% of nights, across study phases for IOA purposes.

#### Sleep Problem Severity Scores

Sleep Problem Severity (SPS) scores were calculated based upon diary data, and video data when diaries were incomplete (i.e., missing information required to determine the quantity of a sleep variable such as SOL and NWs). The SPS score is a composite severity score calculated for each study phase per child (range 0–22), based on averaging their SPS scores for the final seven nights of baseline and of intervention phases, and for all nights in both follow-up periods. A score greater than two is indicative of a clinical level sleep disturbance, i.e., a strong disturbance in one sleep variable, or moderate disturbance in at least two sleep variables (e.g., SOL, CCs, NWs, EMW). Scoring criteria are provided in (McLay et al., 2021; Woodford et al., 2022).

#### Children’s Sleep Habits Questionnaire

The Children’s Sleep Habits Questionnaire (CSHQ; Owens et al., 2000) is a 45-item, parent-report measure used to assess sleep difficulties in 4–10-year-old children. The scores of 33/45 items are summed to obtain a total sleep disturbance score, with scores > 41 indicating clinically significant sleep disturbance based on a neurotypical sample (score range: 33–99). The CSHQ was completed by parents at pre- (assessment phase) and post-intervention (maintenance phase). One participant (Carl) was outside the CSHQ age range; however, it was still administered for consistency, and has been used in prior studies with adolescents (e.g., Moss et al., 2014; Woodford et al., 2022). The CSHQ has strong psychometric properties (e.g., sensitivity [0.80]; specificity [0.72]; internal consistency [α = 0.68–0.78]; and test–retest reliability [*r* = 0.62–0.79]; Owens et al., 2000). Although not validated for use with children with RGNC, it is the most widely used standardized measure of sleep disturbance for neurodiverse populations (e.g., Lambert et al., 2016; McLay et al., 2021; Moss et al., 2014).

#### Treatment Acceptability

The Treatment Acceptability Rating Form—Revised (TARF-R; Reimers et al., 1992) is a 20-item, parent-report measure of intervention satisfaction, given to parents to complete during the maintenance phase. It has six subscales: Reasonableness, Effectiveness, Cost, Willingness, Negative Side-Effects and Disruption/Time. Ratings from 17/20 items (i.e., those related to acceptability) are summed to provide a total acceptability score. Higher scores (maximum = 119) indicate greater acceptability.

#### Treatment Fidelity

Treatment fidelity was calculated by comparing the components of each child’s intervention protocol with procedures noted in diaries across intervention phases (aim > 30% of nights), using the formula (number of intervention components implemented/total recommended components) × 100.

#### Interobserver Agreement

IOA was calculated by comparing diary and video data for participants across study phases (aim > 20% of nights). IOA was only evaluated where behaviors were detectable by parents, so was omitted for Carl. Agreement about duration (e.g., duration of NWs, SOL) required video and diary data to agree within ± 15 min. Percentage of agreement was calculated using the formula [Agreement/(Agreement + Disagreement)] × 100.

### Procedure

#### Clinical Interview

Each family completed a clinical interview, conducted by a licensed Psychologist. The Sleep Assessment Treatment Tool (SATT; Hanley, 2005) was used to guide interviewing around sleep. Demographic information, information about the family context, and the child’s developmental history were also gathered.

#### Functional Behavioral Assessment (FBA) and Behavioral Case Formulation

FBA was completed for each child using data from the clinical interview, sleep diaries, and a minimum of one night of video recording. Then, behavioral case formulation and intervention planning occurred (Blampied, 2013; see Table 2 for FBA outcomes). The guided participation model (Sanders & Burke, 2014) was used to establish parent-clinician consensus regarding the case formulation, intervention goals, the sequential structure of the intervention (using the framework of the less restrictive ordering described above), as well as to promote parental self-efficacy about their intervention (France, 2022).

**Table 2:** Sleep difficulties, factors precipitating and maintaining the sleep difficulties, hypothesized function, and interventions implemented across participants (participants ordered by baseline length)

Participant	Allocated baseline period	Sleep problem type	Factors precipitating or maintaining sleep problem	Hypothesized function	Phase 1: Circadian modifications	Phase 2: antecedent modifications + positive reinforcement	Phase 3: modified extinction
Courtney	1 wk	SOL, NWs, sleep setting, bed-sharing	Insufficient sleep pressure; sleep setting; parent presence	Social attention	Sleep rescheduling (bedtime 9 pm, waketime 7am)	Changes to the sleep setting (couch to own bed)	Modified extinction (+ social story)
Liam	1 wk	SOL, CCs, NWs, bed-sharing	Insufficient sleep pressure; parent presence	Social attention; tangible (light switch, curtains)	Sleep rescheduling (bedtime 8.30 pm, waketime 6.30am)	N/A	Modified extinction
Greg	1 wk (1 day extension)	SOL, NWs, parental presence	Insufficient sleep pressure; parent presence	Social attention; tangible (earmuffs)	Sleep rescheduling (bedtime 9 pm, waketime 6.15am)	N/A	Modified extinction
Carl	1 wk (3-day extension)	SOL, NWs, EMW	Insufficient sleep pressure	Tangible (toys for EMW)	Faded bedtime (9.30 pm – 9.15 pm – 9 pm) + consistent wake time (6.30am)	N/A	N/A
Polly	2 wk	SOL, CCs, NWs	Insufficient sleep pressure; inconsistent sleep schedule	Social attention; escape	Faded bedtime (9 pm – 8.45 pm – 8.30 pm) + consistent waketime (7am – 6.30am)	Set reading time before bed	N/A
Henry	2 wk (3-day extension)	SOL, NWs, parental presence, EMW	Insufficient sleep pressure; inconsistent sleep schedule; parent presence	Social attention; tangible (light switch and toys for EMW)	Sleep rescheduling (bedtime 8 pm, waketime 6am) + elimination of naps	Light switch cover + social story + rewards	Modified extinction
Emily	3 wk	EMW	Insufficient sleep pressure; access to items	Tangible (I-pad & food)	Faded bedtime + consistent waketime (6am)	Social story + Gro-clock	N/A
Jack	3 wk	SOL, NWs, bed-sharing	Insufficient sleep pressure; parent presence	Social attention; tangible (pacifier)	Sleep rescheduling (bedtime 9 pm, waketime 7am)	Social story + Gro-clock + rewards	Modified extinction

Pseudonyms used

*EMW* Early morning waking, *NWs* Night wakings, *SOL* Sleep onset latency, *wk* week

#### Baseline

Participants were randomly allocated a baseline length of 7, 14, or 21 days, although this was extended for three participants due to extraneous factors (e.g., illness) or unstable baseline trends (Barlow et al., 2009). During baseline, parents were instructed to continue implementing their normal routine and respond to their child’s behavior as they typically would.

#### Intervention

Intervention components, which commenced immediately following baseline and were implemented sequentially thereafter for each participant, are outlined in Table 2. Strategies from earlier phases continued in subsequent phases. The decision to move to the subsequent phase was made when no improvements, or insufficient improvements had been evident, and data was stable for a minimum of three days in the current phase. Assessment was repeated as necessary to determine subsequent phases. Intervention continued until the family’s goals had been met and/or the sleep disturbance had resolved for a minimum of 7 nights. For one participant (Emily) melatonin was also gradually faded at the end of the intervention, at the request of the family and in consultation with her physician.

#### Intervention Phase One: Circadian Modifications

To address insufficient sleep pressure (often manifesting in delayed SOL and persistent NWs) and inconsistent sleep schedules, circadian modification was first implemented for all participants. This included elimination of daytime naps (one participant), changes to bed and/or waketime (i.e., sleep/wake rescheduling; five participants) and faded bedtime procedures (Piazza et al., 1997; Schreck, 2022; three participants). Faded bedtime procedures involved initially delaying the child’s bedtime to the average time of sleep onset during baseline, and then moving it earlier in 15-min increments when SOL was consistently (three consecutive nights) < 15 min. This continued until the parents’ developmentally appropriate goal bedtime was met. A consistent waketime was also negotiated with the family and set as part of this procedure.

#### Intervention Phase Two: Antecedent Modifications and Positive Reinforcement

Antecedent modifications were required for five participants and included removal of the bedroom light (one participant), scheduled pre-bedtime reading (one participant), Social Stories™ (i.e., an individualized, descriptive story of the desired night-time routine and behavior; Gray, 2010; four participants) and/or a Gro-clock™ (i.e., a clock used to visually depict bedtime and waketime; two participants). Positive reinforcement for sleep-facilitative behavior was also included alongside antecedent modifications for two participants but not otherwise used, based on parents’ preferences and/or limited child capacity to understand and cooperate with the reinforcement procedure.

#### Intervention Phase Three: Modified Extinction

To address ongoing difficulties with unwanted bed-sharing and parental involvement following sleep-interfering behavior, extinction modified with faded parental presence (see Carnett & McLay, 2022) was implemented for five participants. Although parent–child social interaction during SOL and NW was removed (the extinction component), parents remained within view, systematically increasing their distance from the child (the fading procedure; France & Blampied, 2005; Carnett & McLay., 2022). A Social Story was first introduced to assist with implementation of a modified extinction procedure for one participant (Courtney) to help communicate how and why their parent was fading their presence, and the child’s role in the process.

#### Maintenance and Follow-up Periods

Following intervention, participants entered a maintenance phase during which parents were advised to continue implementing the final intervention conditions, but researcher-initiated contact and nightly data collection ceased. Then, STFU and LTFU data were collected for one week, 4–6 weeks (*M* = 4.9) and 10–14-weeks (*M* = 12.3) post-intervention, respectively. Parents also completed questionnaires and an interview with an independent interviewer.

### Data Analysis

#### Visual Analysis

The sequential effectiveness of each intervention, judged phase-by-phase as it was applied across participants, was evaluated using visual analysis (Parsonson & Baer, 1978). Data from sleep diaries and video (in cases which had missing diary data) were graphed using SigmaPlot 14 software (systatsoftware.com). To supplement visual analysis, the Percentage Below the Median (PBM; Parker et al., 2011) was computed for each intervention and follow-up phase across participants using the baseline median per dependent variable. The baseline median of two participants (Polly and Henry) was zero for the duration of NWs and EMW, despite parents reporting it as a problem and both having five baseline nights where it was notable. To prevent non-occurrences skewing the results, for these cases the baseline median was calculated from those nights where the behavior was a problem, rather than every baseline night. In accordance with PBM criteria, a PBM > 90% represents high intervention effectiveness; 70%–90% represents moderate effectiveness; and < 70% represents an ineffective intervention (Ma, 2006).

#### Modified Brinley Plots

Modified Brinley plots, a form of scatterplot which displays individual participant data within the context of a group (Blampied, 2017), were used to assess change in CSHQ and SPS scores relative to baseline at post- intervention and follow-up phases. Participants’ data at any subsequent time point (e.g., end of intervention) are plotted on the Y-axis against their baseline scores plotted on the X-axis. A 45° diagonal line (i.e., where X = Y) represents no-change. Data points above or below this line indicate a decrease or increase in scores respectively. Additional lines which represent the measure’s clinical cut-off are also displayed.

#### Effect Size Calculation

The within-subjects Cohen’s *d*_*av*_ (Cohen, 1988; Lakens, 2013) and the Percent Superiority Effect Size (PSES; McGraw & Wong, 1992) were calculated for SPS and CSHQ data using software by Cumming (2012) and Lakens (2013). Cohen’s (1988) criteria, namely ≤ 0.2 = small, ~ 0.5 = medium, and ≥ 0.8 = large, were used in part to guide interpretation of results. Negative *d*_*av*_ values represent a clinically desirable reduction in SPS and CSHQ scores from pre- to post-intervention. Confidence intervals (95%CI) about *d*_*av*_ were also calculated (using Cumming, 2012) to assess whether *d*_*av*_ was reliably different from zero (*p* < 0.05). The PSES represents the likelihood a randomly selected participant will have a post-intervention score clinically better than their baseline score (Lakens, 2013; McGraw & Wong, 1992).

## Results

**Fig. 1:**
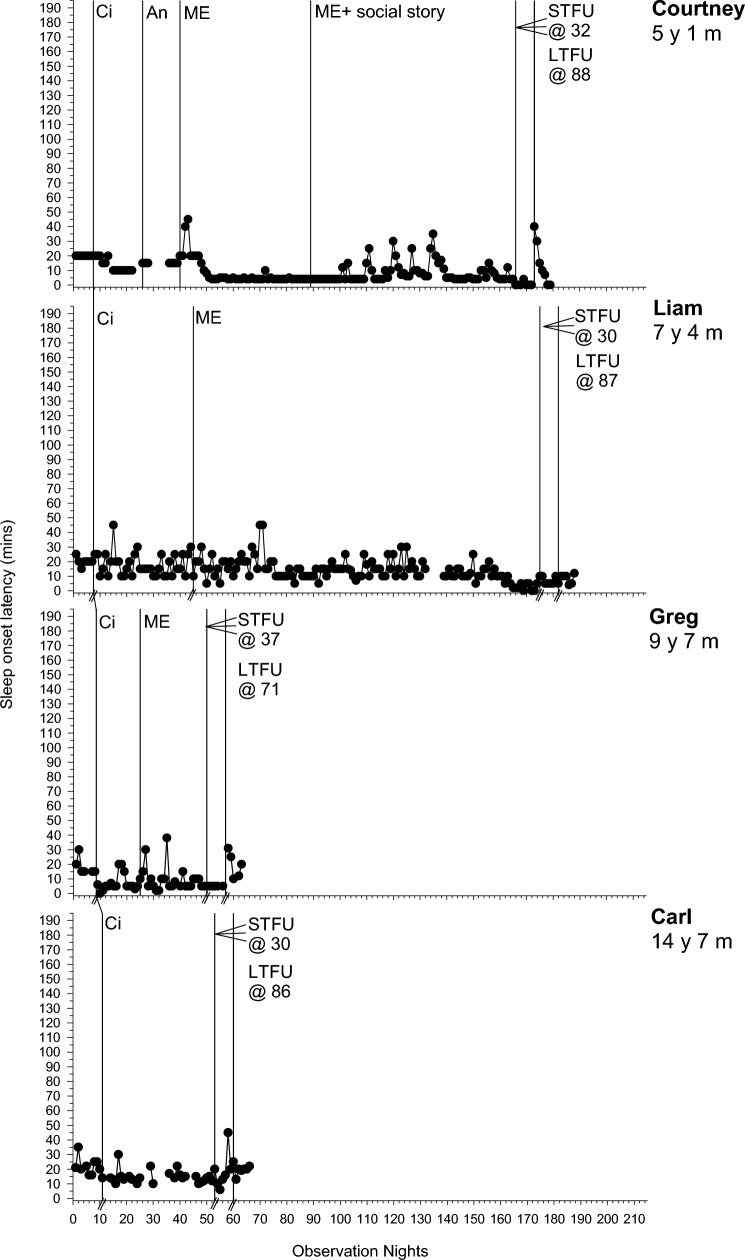

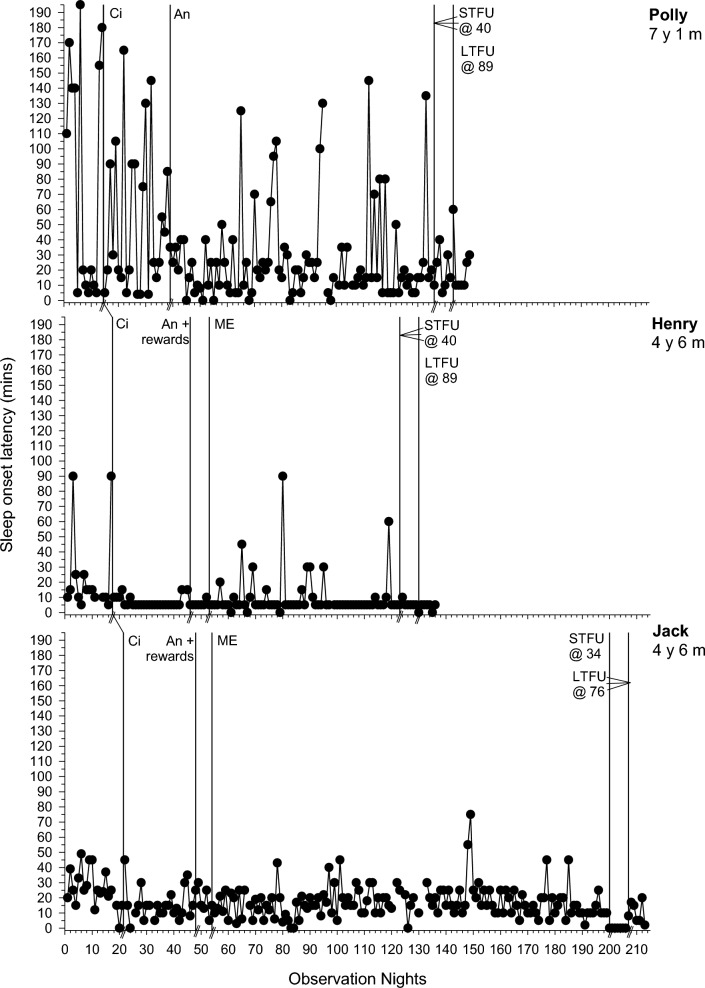
Sleep onset latency (minutes) across participants. *An* Antecedent modifications, *Ci* Circadian modifications, *ME* Modified extinction, *STFU* short-term follow-up, *LTFU* long-term follow-up, @ followed by a number represents the number of days follow-up commenced following intervention

**Fig. 2:**
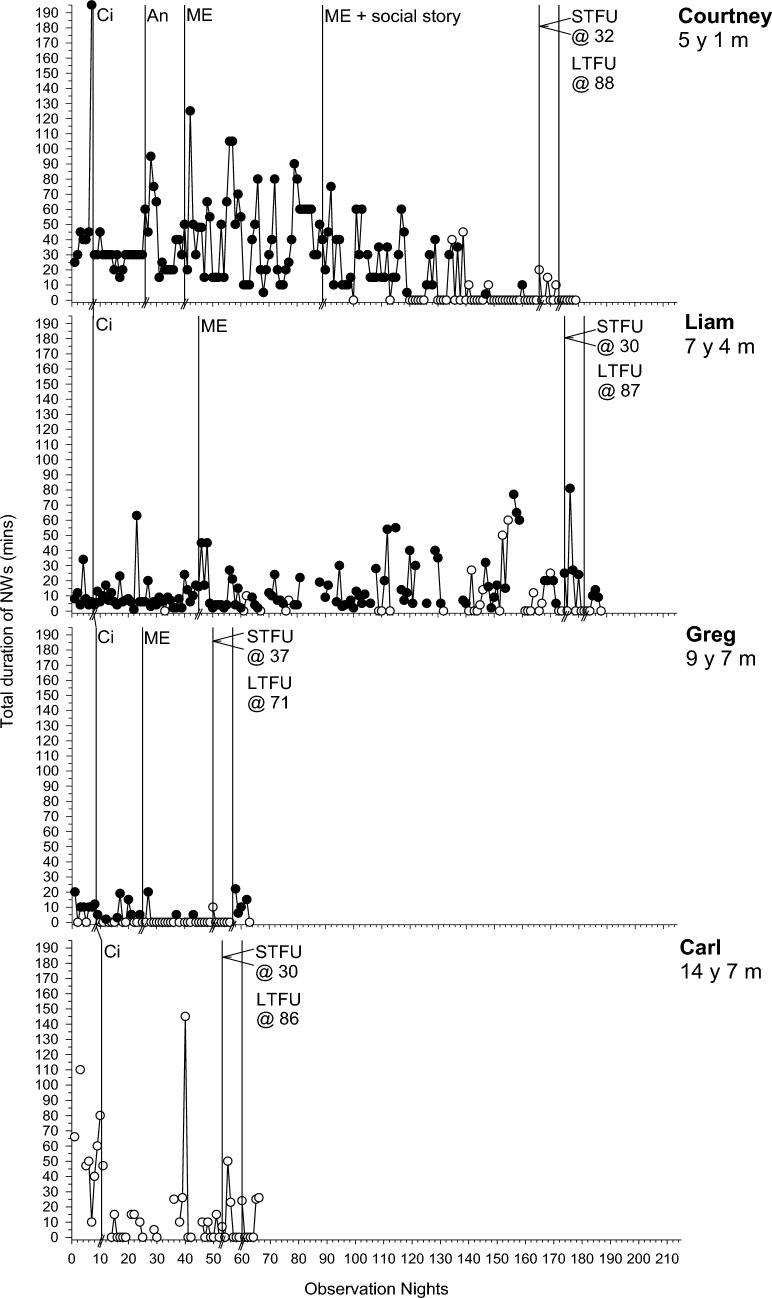

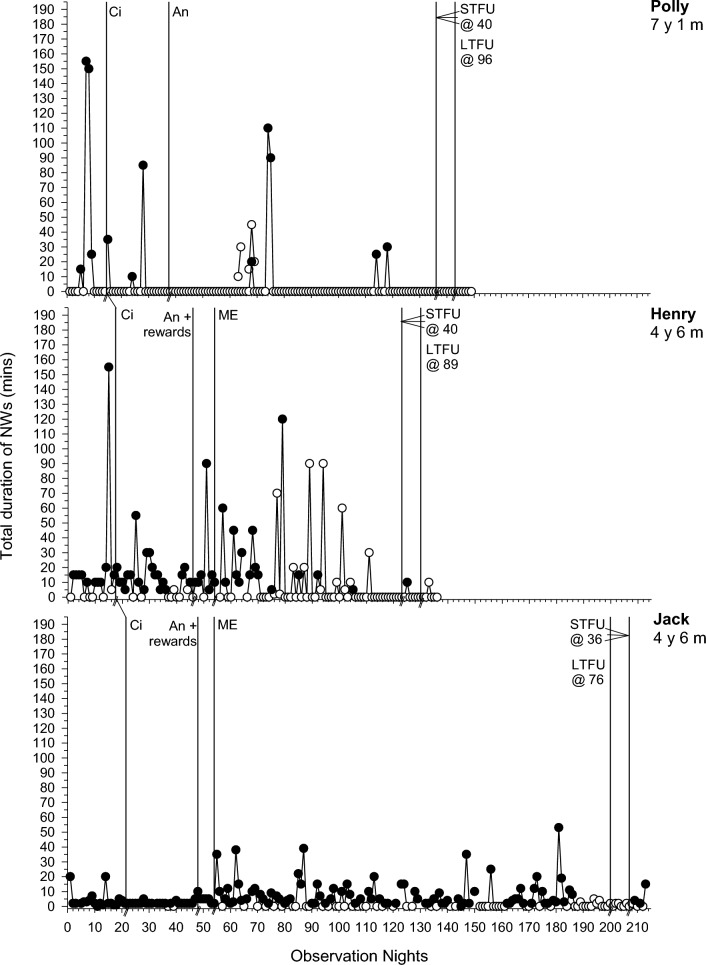
Total duration of night wakings (minutes) across participants. Filled data points = parental presence or involvement; Unfilled data points = no parental presence or involvement. Abbreviations as in Fig. 1

**Fig. 3:**
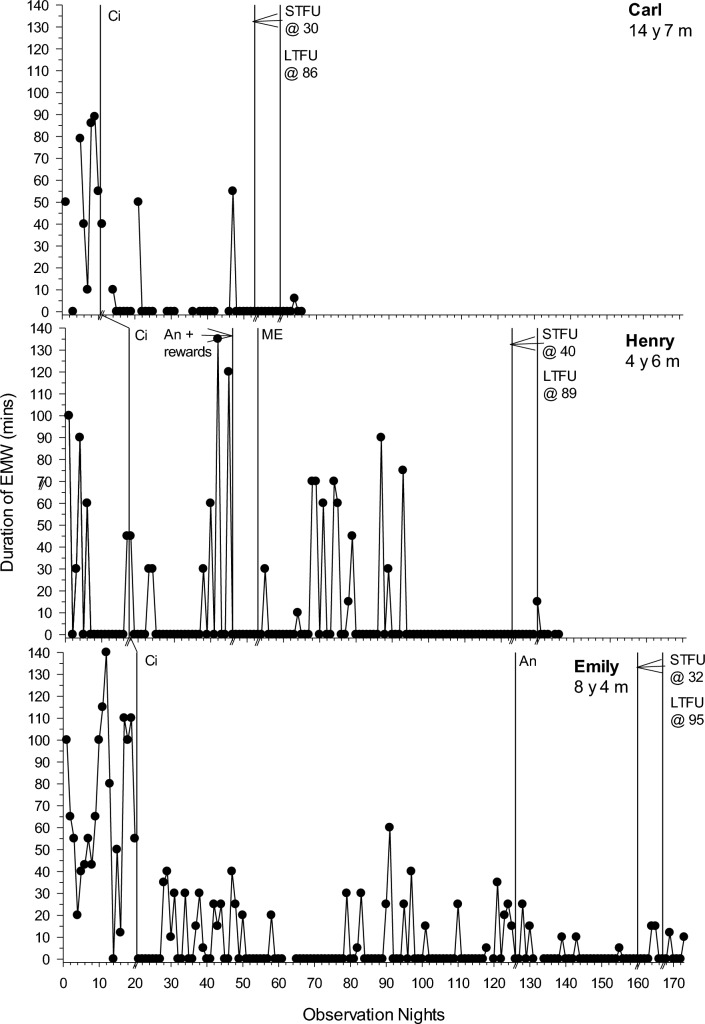
Duration of early morning waking (i.e., minutes awake before the family’s set acceptable waketime) across participants. Abbreviations as in Fig. 1

**Fig. 4:**
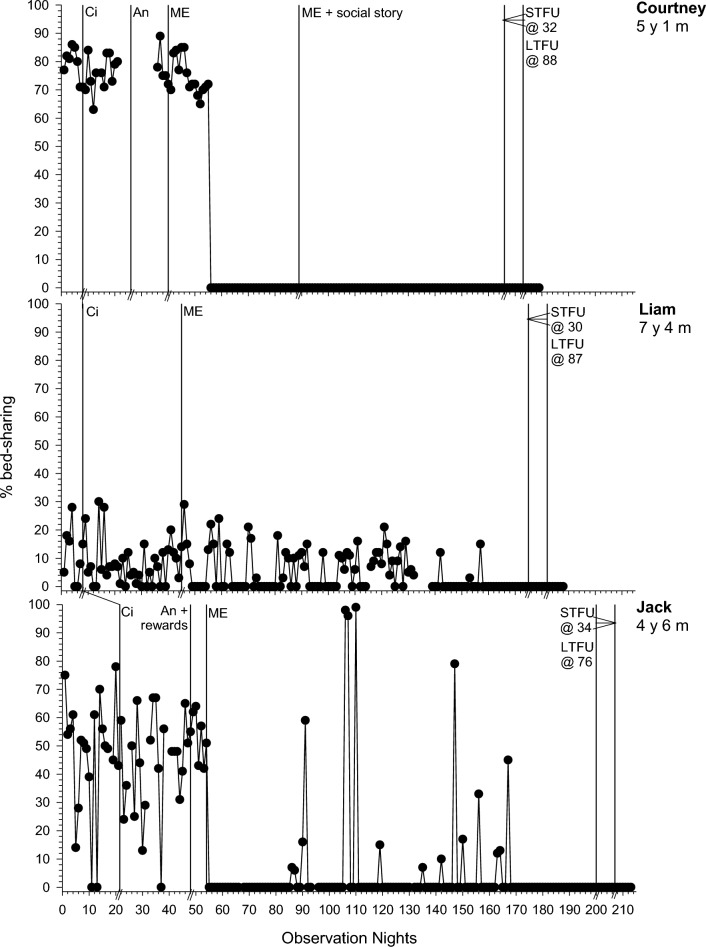
Percentage of the night spent bed-sharing with parents across participants. Abbreviations as in Fig. 1

Following an assessment of baseline variability, level and trend, data are presented across sequential intervention phases for each participant where the dependent variable was deemed an intervention target following FBA and consultation with the parents. There were six dependent variables: SOL (minutes), total duration (minutes) of NWs, frequency of NWs, the duration of EMW (minutes), percentage of the night bed-sharing (total minutes spent bed-sharing/total time spent in bed × 100), and frequency of CCs. The first five variables were graphed (see Figs. 1, 2,3,4 and Online Resource 1). CCs could not be graphed due to lack of specific data in diaries (e.g., exact frequency of CCs), but are referred to in Table 3. Table 3 summarises individual participant PBM results across target dependent variables per phase, while Online Resource 2 depicts the mean PBM results for each dependent variable per phase. Our clinical aspiration was to achieve SOL < 15 min and have no unnecessary night wakings, CCs, bed-sharing or EMW (other than needed for health and safety reasons), but intervention goals were determined in collaboration with parents. Following discussion of behavior change across phases, SPS, CSHQ, IOA, and intervention fidelity and acceptability data are presented.

**Table 3:** Percentage below the median (PBM) calculations per participant for the last week of each intervention phase, and follow-up phases across target dependent variables

Dependent variable	Participant	BL median	PBM last wk of Ci (%)	PBM last wk of An + rewards (%)	PBM last wk of ME (%)	PBM STFU (%)	PBM LTFU (%)
Delayed SOL (mins)	Courtney	20	100	100	100	100	71
	Liam	20	43	N/A	100	100	100
	Greg	15	71	N/A	100	100	60
	Carl	21	100	N/A	N/A	86	71
	Polly	65	71	86	N/A	100	86
	Henry	15	71	100	86	100	100
	Jack	25	71	40	86	100	100
Freq. of CCs	Liam	ND	ND	ND	ND	ND	ND
	Polly	3	71	86	N/A	100	71
Freq. NWs	Courtney	2	0	0	100	100	100
	Liam	5	86	N/A	100	100	100
	Greg	1	57	N/A	86	86	80
	Carl	4	100	N/A	N/A	100	100
	Henry	1	29	33	100	86	86
	Jack	1	0	0	57	43	43
Duration NWs (mins)	Courtney	40	100	75	100	100	100
	Liam	8	29	N/A	43	43	57
	Greg	10	86	N/A	100	86	40
	Carl	53	86	N/A	N/A	100	100
	Polly	87.5	100	100	N/A	100	100
	Henry	10	57	33	100	86	86
	Jack	2	0	0	57	43	43
Duration EMW (mins)	Carl	52.5	86	N/A	N/A	100	100
	Henry	60	57	100	100	100	100
	Emily	60	100	100	N/A	100	100
% Bed-sharing	Courtney	81	57	75	100	100	100
	Liam	8	29	N/A	100	100	100
	Jack	50	71	20	100	100	100

Pseudonyms used

*An* Antecedent modifications, *BL* Baseline, *Ci* Circadian modifications, *EMW* Early morning waking, *LTFU* Long term follow-up, *Mod extinction* Modified extinction, *N/A* Not Applicable, *ND* No data, *NWs* Night wakings, *PBM* Percentage below the median, *SD* Standard deviation, *SOL* Sleep onset latency, *STFU* Short term follow-up, *wk* week

### Baseline

SOL was an intervention target for seven participants, with the most identified hypothesised function being insufficient sleep pressure. The baseline trends were stable for all (see Fig. 1) although baseline was trending in the therapeutic direction mid-baseline for Polly and Jack, this stabilised permitting visual analysis. NWs were an intervention target for seven participants, with the most identified hypothesised function being social attention (NWs for 6/7 participants resulted in bed-sharing or parental intervention). Hence, NWs were graphed even when short in duration (see Fig. 2 and Online Resource 1). The baseline trends were stable or trending in the non-therapeutic direction for all participants, permitting visual analysis. EMW was an intervention target for three participants (see Fig. 3), with the hypothesised function being access to tangible items (e.g., toys and device) for all. Both Emily and Carl have variable data, but EMW was the primary intervention target. For Henry, EMW was a difficulty at the start of baseline but this stabilised and was no longer problematic at the end; however, his data was still graphed as EMW re-emerged as a problem and became an intervention target at the end of phase one. Bed-sharing was an intervention target for three participants (see Fig. 4). Courtney showed a stable trend, Liam a low cyclic trend and Jack a high level of variation, and all were acceptable for visual analysis.

### Phase One: Circadian Modifications

For all participants, phase one involved circadian modifications. SOL improved for 6/7 participants (PBM = 43%–100%; Fig. 1). The frequency and duration of NW improved for 2/6 (Liam and Carl; PBM = 86% and 100% respectively) and 3/7 (Polly, Greg and Carl; PBM = 86–100%; Fig. 2) participants respectively. EMW improved and practically resolved for 2/3 participants (Carl and Emily; PBM = 86% and 100% respectively), indicating a strong intervention effect for that behavior (Fig. 3). The third participant (Henry) displayed an immediate effect; however, this was not maintained during this phase (PBM = 57%). Results were mixed for bed-sharing (PBM = 29%–71%), with no or a moderate intervention effect (Fig. 4). All participants except Carl then moved into a further intervention phase. Two (Liam and Greg) transitioned straight from phase one to three based on the determined function of the sleep difficulty (parental attention), parent preference and/or limited child capacity to understand and cooperate with reinforcement procedures and Social Stories.

### Phase Two: Antecedent Modifications and Positive Reinforcement

Five participants (Courtney, Polly, Henry, Emily and Jack) next experienced antecedent modifications, plus positive reinforcement for Jack and Henry. There was further improvement in SOL in phase two for 2/4 participants (Polly and Henry; PBM = 86% and 100% respectively; Fig. 1). This was the same for Polly in relation to CCs (PBM = 86%). There was no further improvement for NWs (PBM = 0%-33%; Fig. 2). Although all participants with problems with EMW exhibited clear improvements after circadian modifications, these were not maintained (Fig. 3). Henry showed a deterioration after several weeks, where he would wake early and turn the bedroom light on. This immediately resolved in phase two following introduction of a light switch cover and Social Story (PBM changed from 57 to 100%). Emily also showed a slight deterioration as her bedtime was brought forward using the faded bedtime procedure. The addition of a Social Story and Gro-clock resolved this (PBM = 100%). This removed the need for a modified extinction procedure (i.e., removal of a device). For those where bed-sharing was a problem, improvements were noted for only one participant (Courtney; PBM increased from 57 to 75%; Fig. 4), with changes to her sleep setting (couch to own bed) and a subsequent improvement in NWs.

### Phase Three: Modified Extinction

Five children (Courtney, Liam, Greg, Henry and Jack) required a modified extinction procedure specifically targeting withdrawal of parent attention, to resolve bed-sharing (PBM = 100%) and parent involvement following NWs. Liam gradually reached 0% bed-sharing in the final two weeks of intervention (PBM = 100%). Courtney and Jack exhibited a sudden change in bed-sharing due to procedural changes (e.g., Courtney’s parent used a mattress rather than bed-sharing and Jack’s parent started returning Jack to his own bed; Fig. 4). There were further improvements in SOL for the three participants where SOL had not yet resolved (Liam, Greg and Jack PBM = 86%–100%; Fig. 1). There were also improvements in the frequency (PBM = 57%–100%; Jack exhibited a floor effect) and duration of NWs (PBM = 43%–100%; Liam and Jack exhibited a floor effect) for all participants where it remained a problem (Fig. 2). For Courtney, a clear reduction was evident after the addition of a Social Story, which although not a planned part of this phase, was added to facilitate the procedure as decided with the parents. There was some fluctuation in SOL and NWs throughout this phase (i.e., response bursts), which as expected, was associated with reductions in parent responses as part of modified extinction procedures or child illness. Modified extinction was not needed to improve EMW.

### Follow-up

Improvements maintained at STFU and LTFU for SOL (PBM = 60%–100%; Fig. 1), CCs (PBM = 100%), frequency and duration of NWs (PBM = 40%–100%; Fig. 2), EMW (PBM = 100% for all; Fig. 3) and bed-sharing (PBM = 100% for all; Fig. 4) for most participants, except for Henry and Greg who showed a deterioration. There was some variation at LTFU, particularly for NWs. For Liam, NWs remained a difficulty at the end of intervention despite consistent implementation of strategies, nevertheless the maintenance phase began with parental agreement. NWs improved further and stabilised during that phase.

### Summary of Results Across Phases

Circadian modifications had the greatest impact on difficulties related to sleep onset (SOL) and offset (EMW), compared to other sleep difficulties. Further improvements in SOL and EMW were observed with the introduction of antecedent modifications, but these had little impact on behaviors which precipitated parental involvement including NWs and bed-sharing. Night-time parental involvement was resolved for all participants only with modified extinction. For some, NWs persisted but they were lower in frequency and duration, and no longer caused concern to parents.

### Sleep Problem Severity (SPS)

**Fig. 5:**
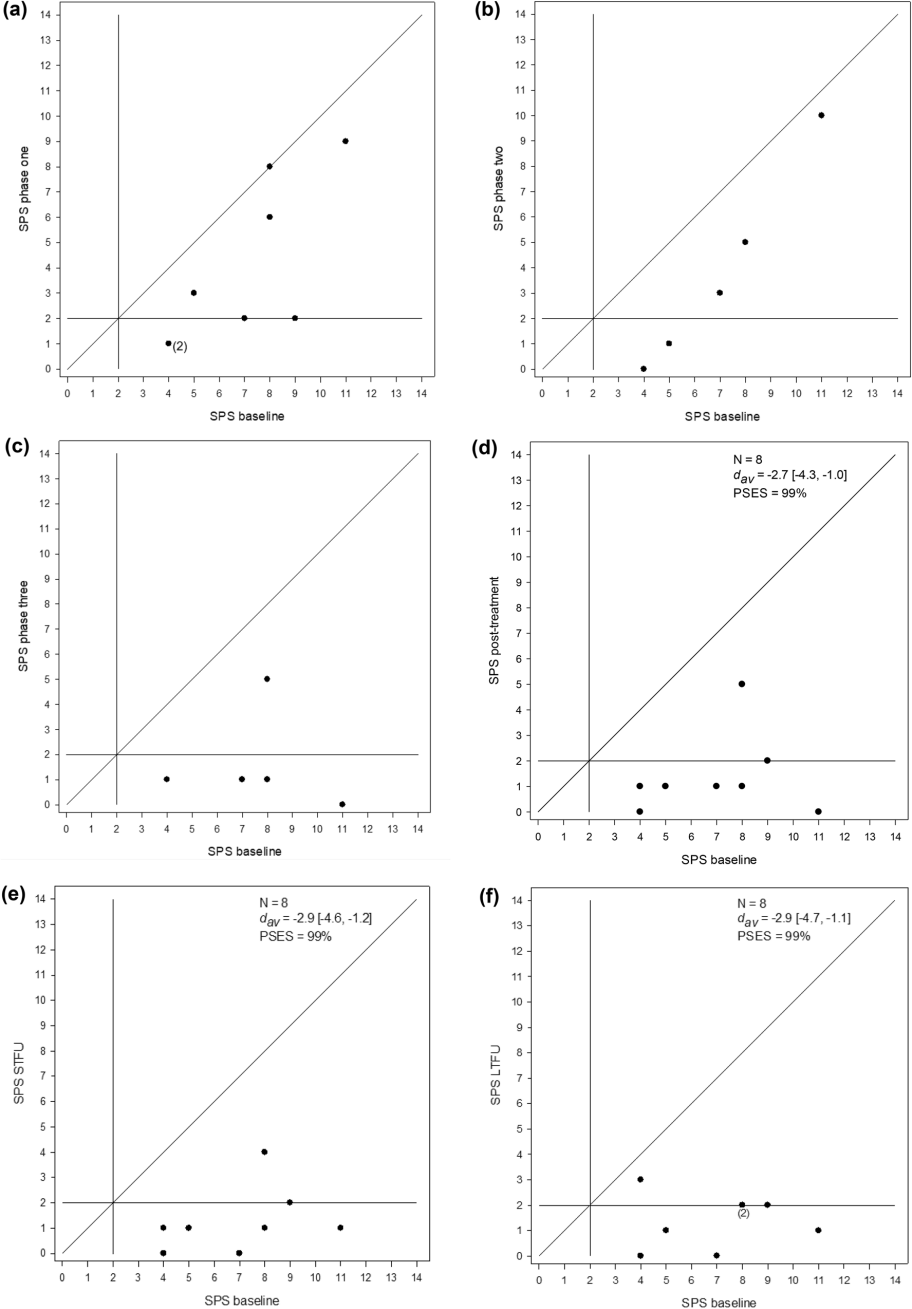
Modified Brinley plots showing participant change in sleep problem severity (SPS) scores from baseline to phases one, two and three, the last phase of intervention, short-term (STFU) and long-term follow-up (LTFU)

Participant SPS scores across phases are shown in Table 4, and changes in SPS scores from baseline to all intervention phases, the final week of intervention, and follow-up periods are shown in Figs. 5a–f. Mean SPS scores reduced with each phase. The effects were large and statistically significant (i.e., 95% CI do not cross zero) from baseline to the final phase of intervention (*d*_*av*_ = − 2.7; 95% CI = − 4.3 to − 1.0; PSES = 99%), STFU (*d*_*av*_ = − 2.9; 95% CI = − 4.6 to − 1.2; PSES = 99%) and LTFU (*d*_*av*_ = − 2.9; 95% CI = − 4.7 to − 1.1; PSES = 99%).

**Table 4:** Sleep problem severity (SPS) scores per participant for the last week of baseline, sequential intervention phases, and follow-up phases across participants (ordered according to baseline length)

Participant	SPS BL	SPS Ci	SPS An + rewards	SPS ME	SPS STFU	SPS LTFU
Courtney	11	9	10	0	1	1
Liam	8	8	N/A	5	4	2
Greg	4	1	N/A	1	1	3
Carl	9	2	N/A	N/A	2	2
Polly	5	3	1	N/A	1	1
Henry	7	2	3	1	0	0
Emily	4	1	0	N/A	0	0
Jack	8	6	5	1	0	0
*Mean* *SD*	7 2.5	4 3.2	3.8 4.0	1.6 1.9	1.3 1.3	1.4 1.1

Pseudonyms used

*An* Antecedent modifications, *BL* Baseline, *Ci* Circadian modifications, LTFU = Long term follow-up, ME = Modified extinction, N/A = Not Applicable, SD = Standard deviation, SPS = Sleep problem severity score, STFU = Short term follow-up

### Children’s Sleep Habits Questionnaire

All participant CSHQ scores reduced post-intervention (pre- *M* = 55.1, SD = 6.3; post- *M* = 45, SD = 6.3), with a large and statistically significant effect (*d*_*av*_ = − 1.61; 95% CI = − 2.6 to − 0.59; PSES = 95%). Post-intervention scores fell on or below the clinical cut-off for three participants. A figure showing change in CSHQ scores from pre- to post-intervention is in Online Resource 3.

### Treatment Acceptability

Eight mothers and five fathers completed the TARF-R, with total scores ranging from 81 to 119 (*M* = 104.5). Average ratings yielded higher scores in the Effectiveness (19.8/21) subscale, and lower scores in the Disruption (15.9/21) and Side Effects (17.6/21) subscales. See Online Resource 4 for full TARF-R results.

At least one parent per child also completed a post-intervention interview. Five noted having difficulty consistently implementing the set sleep/wake schedule, however, 3/5 reported this was the strategy they believe had the greatest impact. All five who had completed modified extinction also noted challenges maintaining consistent responses but reported the rationale behind the process was clear. Child illness, COVID-19 lockdowns, family holidays, and varying family factors were reported to impact intervention. For 5/8 participants parents reported at least two periods (minimum 3 days) of illness during which intervention was on hold. Such factors, in combination with the staggering of intervention components influenced the total duration of intervention, which was prolonged for some (*M* = 146 nights; range = 63 – 213 nights including baseline). Despite challenges, all reported that the outcomes achieved were worth the effort. Although changes following antecedent modifications were not notable for most participants, three parents spoke highly of the use of Social Stories and Gro-clock which they felt gave their children autonomy and helped them to understand changes. All parents commented on the benefit of having close, individualised clinical support throughout.

### Treatment Fidelity

Treatment fidelity was calculated for 32–62% (*M* = 36%) of nights across intervention phases and was high for intervention (*M* = 83%, range 67–100%) and STFU (*M* = 91%, range 67–100%), but lower for LTFU (*M* = 70%, range 29%–100%) and satisfactory overall (*M* = 82%, range = 63–100%).

### Interobserver Agreement

IOA data were able to be calculated for 7/8 participants across study phases and for 19%-34% (*M* = 21%) of nights across phases. Mean IOA was 91% (range, 84%–98%) across all phases and behaviors.

## Discussion

This study firstly evaluated the practicality of combining FBA-informed intervention planning with the principle of least restriction and while using the guided participation model with parents. Secondly, it evaluated the effectiveness and acceptability of behavioral sleep interventions for children with RGNC, where interventions were sequenced from less to more restrictive to ascertain whether any were minimally sufficient for individual children, or across this group of children. Results suggested the less restrictive strategy of circadian modification was particularly effective in reducing SOL and EMW, relative to other sleep difficulties. Other antecedent modifications and positive reinforcement strategies resulted in further improvements across sleep difficulties, except for NW and unwanted bed-sharing. These difficulties required a modified extinction procedure. This study demonstrated that FBA could be successfully combined with the least restrictive principle, and overall, behavioral interventions were effective at reducing sleep difficulties across all participants. Improvements maintained at follow-up and were rated favorably by parents.

Consistent with previous research, difficulties with initiating and maintaining sleep (e.g., delayed SOL, NWs and EMW) were the most reported type of sleep difficulty (Angriman et al., 2015; Kronk et al., 2010; Moss et al., 2014). A range of factors were identified as likely underpinning these difficulties. A lack of homeostatic sleep pressure related to developmentally inappropriate and/or inconsistent sleep/wake schedules was common. Behavioral factors included inappropriate and inconsistent discriminative stimuli for sleep (e.g., variation in sleep settings, light cues, and routines) and contingencies of reinforcement (e.g., parental attention and tangible items). Other contributing etiological factors could be neurobiological (e.g., circadian irregularities; Woodford et al., 2021), although these were not assessed.

It appears each intervention component played a qualitatively different role, targeting different types of sleep disturbance. Circadian modifications were implemented for all participants where insufficient sleep pressure as a result of developmentally inappropriate total sleep duration and inconsistent sleep/wake schedules was perceived to play a role. They were effective in reducing SOL, NWs and EMW for most participants, likely due to an increase in homeostatic sleep pressure (Piazza et al., 1997). This was a minimally sufficient intervention for one participant, but in most cases was not sufficient to resolve the sleep difficulty. The addition of antecedent modifications further improved sleep difficulties such as SOL and EMW for most. It is unclear, however, whether this is due to prolonged implementation of circadian modifications, or the additive effect of antecedent and/or positive reinforcement strategies, or both. Given data stability was required before transitioning to subsequent phases, it seems more likely for the latter to be the case. In one case a combined faded bedtime + Gro-clock + Social Story fully eliminated the need for removal of tangible items (e.g., food and device). In addition to increased sleep pressure, these improvements may be attributed to antecedent changes including clear rules around bedtime behavior and consistent discriminative stimuli for sleep onset and offset (Blampied & Bootzin, 2013; Woodford et al., 2022). These findings suggest less restrictive options should be tried first in the treatment of sleep disturbance, to minimise the need for extinction procedures.

Modified extinction was required, however, for all five participants where the function of the sleep difficulty was parent attention (most commonly NWs and unwanted bed-sharing). In these cases, faded parental presence was used to minimise the intensity of response bursts and proceed at the preferred pace of the parents (Carnett & McLay, 2022). Given that preceding circadian and antecedent modifications and positive reinforcement resulted in some improvement in NWs, this likely facilitated subsequent change and reduced the impact of modified extinction in two ways. First, increased sleep pressure may have reduced opportunities and/or motivation for child protesting during the night. Second, antecedent modifications such as Social Stories meant the children understood expectations around night-time, which may have resulted in them being more amenable to change.

Intervention effects were maintained long-term for most participants. The one exception was likely associated with poor treatment fidelity during the maintenance phase. At the request of one family, they were guided to fade out melatonin use and improvements were maintained once melatonin use ceased, suggesting behavioral interventions were sufficient.

Research evaluating the acceptability of behavioral interventions for children with RGNC is limited (Woodford et al., 2022). In this study parents of such children reported behavioral interventions to be acceptable, understandable, and effective. Not surprisingly, parents noted a level of disruption and side effects, mostly related to maintaining strict bed- and wake-times and managing modified extinction procedures. Given this, it is possible sleep hygiene, visual prompts and stimulus control techniques, as opposed to circadian modification, are the least restrictive options for parents, but this might vary case-by-case and may not be the least restrictive options as experienced by children. Further, the challenges experienced might also reflect the level of complexity in managing sleep disturbance in children with RGNC (France et al., 2022). Many participants experienced periods of illness, which disrupted intervention progress in all cases, as intervention was suspended. Despite such factors, parents reported that improvements in sleep were worth the effort.

### Clinical Implications

Important clinical implications of these findings are, first, that many of the participants in this study had co-occurring conditions and periods of illness during intervention but still achieved positive results, suggesting medical complexity need not be a deterrent in treating sleep disturbance. Further, although there is evidence of disrupted melatonin regulation in children with RGNC (Woodford et al., 2021), sleep difficulties can still be addressed using behavioral strategies. Second, there appear to be options that will improve children’s sleep to some extent, before considering modified extinction. Third, based on spontaneous comments made by parents in the post-intervention interview, parents had varying views on what were the least intrusive and stressful interventions, highlighting the importance of working in collaboration with families (guided participation) to determine this on a case-by-case basis. Fourth, with careful planning and implementation, modified extinction procedures are feasible for children with RGNC and are particularly important to consider for treating unwanted bed-sharing. Finally, it is noteworthy that behavioral sleep interventions can be successfully administered through telehealth, as was done in this study. This has implications in reducing barriers to accessing health care.

## Limitations and Conclusions

Limitations include, first, that consistency and universality regarding what is least restrictive for children and their parents is not yet established. Future research should gather this information and evaluate sequentially implemented intervention components accordingly, to deepen our understanding of both the theoretical and clinical aspects of behavioral sleep intervention. Second, the procedure overall required considerable clinical support, which may not always be as readily available as it was in this research study. This has implications for the general clinical utility of such interventions. Third, although an intervention effect was evident, the contributions of antecedent and consequence modifications is unclear given they were administered following circadian modifications. Therefore, conclusions regarding any apparent causal effect need to consider the whole sequence of intervention components. Robust analysis of individual intervention components or individual least restrictive strategies would be beneficial. Finally, the number of replications was moderate (*N* = 8), and the degree to which the children and families in this study are representative within the suite of varying RGNC is unknown. Research with systematic and clinical replication is needed to establish best practice for sleep interventions for children with RGNC (McLay et al., 2019; Woodford et al., 2022). Evaluation of the impact on a range of secondary child, parent, and sibling outcomes (e.g., health, behavior, wellbeing, sleep), as well as the impact of child and family complexity on intervention response would also be beneficial and enhance generalizability (France et al., 2022). Nonetheless, this study is an important starting point in exploring what are the least restrictive and minimally sufficient behavioral sleep interventions for children, which is particularly important for this population who experience a range of co-occurring complexities.

## Supplementary Information

Below is the link to the electronic supplementary material.

Supplementary file1 (DOCX 87 KB)

Supplementary file2 (DOCX 43 KB)

Supplementary file3 (DOCX 24 KB)

Supplementary file4 (DOCX 18 KB)
