# Supplementary material for: The Lighter Touch: Less-Restriction in Sequentially Implemented Behavioral Sleep Interventions for Children with Rare Genetic Neurodevelopmental Conditions
Source: J Autism Dev Disord. 2024 Feb 7;55(2):547–68. doi: 10.1007/s10803-024-06234-4 (PMC11813967; doi:10.1007/s10803-024-06234-4)
Supplement: Supplementary file 1 — Supplementary file1 (DOCX 87 KB) [file 10803_2024_6234_MOESM1_ESM.docx]

**Online Resource 1**

*Frequency of night wakings across participants*

*Note.* An = Antecedent modifications; Ci = Circadian modifications; ME = Modified extinction; STFU = short-term follow-up; LTFU = long-term follow-up; @ followed by a number represents the number of days follow-up commenced following intervention.
