# Supplementary material for: The Lighter Touch: Less-Restriction in Sequentially Implemented Behavioral Sleep Interventions for Children with Rare Genetic Neurodevelopmental Conditions
Source: J Autism Dev Disord. 2024 Feb 7;55(2):547–68. doi: 10.1007/s10803-024-06234-4 (PMC11813967; doi:10.1007/s10803-024-06234-4)
Supplement: Supplementary file 2 — Supplementary file2 (DOCX 43 KB) [file 10803_2024_6234_MOESM2_ESM.docx]

**Online Resource 2**

*Mean percentage below the median results per dependent variable across intervention phases*


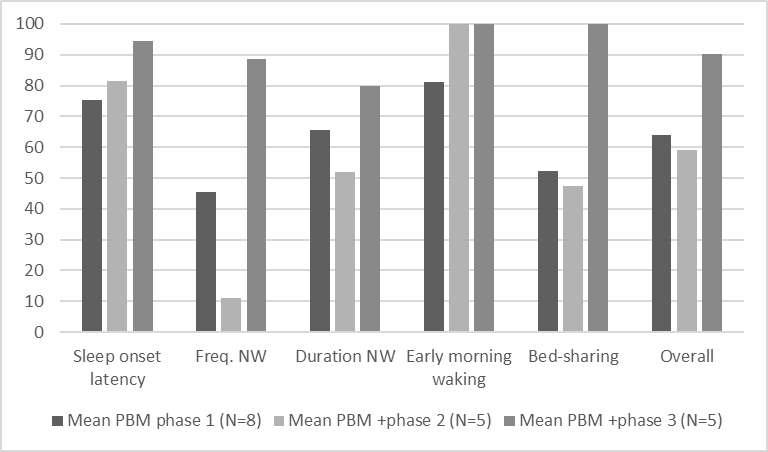


*Note.* Dotted line = Ma (2006) criteria for moderate intervention effect (>70%)
