# Supplementary material for: The Lighter Touch: Less-Restriction in Sequentially Implemented Behavioral Sleep Interventions for Children with Rare Genetic Neurodevelopmental Conditions
Source: J Autism Dev Disord. 2024 Feb 7;55(2):547–68. doi: 10.1007/s10803-024-06234-4 (PMC11813967; doi:10.1007/s10803-024-06234-4)
Supplement: Supplementary file 3 — Supplementary file3 (DOCX 24 KB) [file 10803_2024_6234_MOESM3_ESM.docx]

**Online Resource 3**

*Modified Brinley Plot showing participant change in Childrens Sleep Habits Questionnaire (CSHQ) total scores from pre- to post-intervention*

CSHQ post-intervention
