# Supplementary material for: The Lighter Touch: Less-Restriction in Sequentially Implemented Behavioral Sleep Interventions for Children with Rare Genetic Neurodevelopmental Conditions
Source: J Autism Dev Disord. 2024 Feb 7;55(2):547–68. doi: 10.1007/s10803-024-06234-4 (PMC11813967; doi:10.1007/s10803-024-06234-4)
Supplement: Supplementary file 4 — Supplementary file4 (DOCX 18 KB) [file 10803_2024_6234_MOESM4_ESM.docx]

**Online Resource 4**

*Participant Treatment Acceptability Rating Form-Revised (TARF-R) subscale and total scores*

| Participant | Reasonable | Effective | Side effects | Disruption | Cost | Willing | Problem severity | Understand | Total |
| --- | --- | --- | --- | --- | --- | --- | --- | --- | --- |
| Carl  Mother  Father | 21  21 | 21  21 | 21  21 | 21  20 | 14  14 | 21  21 | 6  14 | 7  7 | 119  118 |
| Greg  Mother | 20 | 21 | 16 | 13 | 13 | 20 | 11 | 7 | 103 |
| Emily  Mother  Father | 21  21 | 21  18 | 20  17 | 17  17 | 14  11 | 21  19 | 10  12 | 7  7 | 114  103 |
| Liam  Mother  Father | 18  16 | 20  17 | 17  17 | 15  17 | 13  14 | 18  20 | 11  12 | 6  6 | 101  101 |
| Polly  Mother | 16 | 15 | 13 | 7 | 14 | 16 | 8 | 6 | 81 |
| Courtney  Mother  Father | 18  16 | 20  21 | 18  16 | 15  13 | 13  12 | 19  18 | 5  8 | 6  6 | 103  96 |
| Henry  Mother | 21 | 21 | 19 | 15 | 14 | 18 | 10 | 7 | 108 |
| Jack  Mother  Father | 21  15 | 21  20 | 20  14 | 19  18 | 14  14 | 17  18 | 3  8 | 7  7 | 112  99 |
| *Mean*  *SD* | 18.8  2.2 | 19.8  2.0 | 17.6  2.4 | 15.9  3.7 | 13.4  1.0 | 18.9  1.7 | 9.1  3.2 | 6.6  0.5 | 104.5  10.5 |

*Note.* TARF-R were given to parents to complete if they were present and/or involved in the programme. SD=Standard deviation.
